# Lightweight Filter Architecture for Energy Efficient Mobile Vehicle Localization Based on a Distributed Acoustic Sensor Network

**DOI:** 10.3390/s130911314

**Published:** 2013-08-23

**Authors:** Keonwook Kim

**Affiliations:** Division of Electronics & Electrical Engineering, Dongguk University-Seoul, Seoul 100-715, Korea; E-Mail: kwkim@dongguk.edu; Tel./Fax: +822-2260-3334

**Keywords:** mobile vehicle localization, energy-based source localization, wireless sensor network, envelope detector, exponential smoothing filter, acoustic source localization, velocity vector estimator

## Abstract

The generic properties of an acoustic signal provide numerous benefits for localization by applying energy-based methods over a deployed wireless sensor network (WSN). However, the signal generated by a stationary target utilizes a significant amount of bandwidth and power in the system without providing further position information. For vehicle localization, this paper proposes a novel proximity velocity vector estimator (PVVE) node architecture in order to capture the energy from a moving vehicle and reject the signal from motionless automobiles around the WSN node. A cascade structure between analog envelope detector and digital exponential smoothing filter presents the velocity vector-sensitive output with low analog circuit and digital computation complexity. The optimal parameters in the exponential smoothing filter are obtained by analytical and mathematical methods for maximum variation over the vehicle speed. For stationary targets, the derived simulation based on the acoustic field parameters demonstrates that the system significantly reduces the communication requirements with low complexity and can be expected to extend the operation time considerably.

## 1. Introduction

Distributed monitoring of a wireless sensor network (WSN) provides source localization capability over a wide spatial region by using various sensor technologies. The operation modes of sensors can be arranged into active or passive methods for detecting the physical aspects of the external environment. Atmospheric variation generated via target introduction is perceived by the potential sensor technologies which have unique properties in the orientation, range, *etc.* [1]. Based on decay model-dependent/independent approaches, the distributed or centric algorithms precisely localize the position of the source for single and multiple targets [2]. Since the computation and communication proportionally require the power of the deployed nodes, the system performance is mainly evaluated by the power consumption. We note that Web of Science shows that as of February 2013 approximately 30% (242/805) of WSN localization papers contain energy or power as keywords.

With its low complexity sensors and algorithms, the WSN performs the localization task efficiently for an extended time. Acoustic localization is an active research area in WSNs since the node architecture requires simple and inexpensive components. Besides the time and phase of the acoustic signal, energy-based localization algorithms are the preferable selection because the acoustic energy emitted by the sources usually decreases gradually. Therefore, the temporal signal can be digitized at a lower sampling rate compared with the raw acoustic time series for other localization algorithms [3]. The conventional acoustic localizations such as beamforming [4] involve high sampling rates, tight synchronization, and intense computation [5] between the receivers; hence, the algorithm displays limited performance within the WSN configuration.

Acoustic localization efforts in WSN are diverse and classified as network, signal processing, and statistic based algorithms. The network-based schemes mainly utilize the relation, quality, and clustering of inter-node communications for localization [6–11]. The signal processing-based methods dynamically employ array, adaptive, and robust filtering in order to find the source position [12–14]. The statistic-based approaches primarily exploit likelihood estimators for locating the target coordinates [3,15–20]. We observe that certain algorithms have hybrid classification due to their multi-level hierarchical localization. The fundamental acoustic localization works concentrate on the constant monitoring of the sound source regardless of target status; therefore, the stationary sources continuously consume system power without producing any valuable information.

This paper presents a lightweight node architecture for mobile vehicle localization based on a distributed acoustic sensor network. Compared with conventional WSN localization algorithms, the proposed architecture uses reduced communication and computation resources due to the velocity vector dependent output of the system. When the sound source approaches the monitoring node with speed, the node produces an elevated output. On the other case, if the source halts in close proximity, the node output gradually diminishes to zero because the target stays in a fixed position. The conventional localization algorithm generates the node output based on the Euclidian distance from the source, therefore, a closely located source continuously accesses the system without providing further information. The novel system in this paper improves the conventional energy-based (CEB) localization with a proximity velocity vector estimator (PVVE). The location of the source is identified by the collective contour plot derived from the individual WSN node outcomes and an additional tracking algorithm is required for displaying the stationary targets on the screen.

In this paper, the definition of the target is restricted to engine mounted vehicles which generate a consistent acoustic sound in all directions. For vehicle localization, the acoustic sensor is operated in passive manner which only receives the source sound continuously. Numerous papers describe the acoustic properties of the vehicle extensively for the automobile compartment [21], exhaust system [22], tire tread [23], tire/road interaction [24], *etc.* Sandberg in particular provided an insightful overview of the tire/road noise emitted by mobile vehicles under various conditions [25]. Duarte also presented an experimental database of acoustic vehicle signals over a field-deployed WSN at 4,960 Hz sampling rate [20]. The acoustic signature of the vehicle contains various clues of its physical movements useful in estimating speed, range, and number of vehicles [26–29]. The previous works by the author introduced the azimuthal movement detection system for vehicles based on diffraction over a binaural receiver and 3D printed architecture [30]. For simulation purposes, the baseline acoustic data is identical to the previous work and the velocity profile is adapted by using the inverse square law (ISL) which describes the magnitude of the acoustic signal over the distance.

The objectives of the PVVE system are presented in Section 2 with a focus on the system overview. The design factor and method of the PVVE system are presented in Section 3. The ISL, which is the essential theory of the PVVE system, is described to explain the fundamental operation process of the system. The node architecture based on the cascade filters is suggested and analyzed to employ physical aspect of the mobile sound source as well. In Section 4, the optimal parameters in the exponential smoothing filter are obtained by analytical and mathematical methods for maximum variation over the vehicle speed. Finally, a simulation based on the acoustic field parameters is demonstrated for various vehicle movement scenarios in Section 5. Note that the PVVE indicates the cascade filters in the node for velocity vector output and the PVVE system is the entire system including the WSN nodes and base station for collective decisions.

## 2. Objectives

The major goal of the PVVE system is to shorten the node operation time of the acoustic energy-based WSN localization system by understanding the physics of the motion. The collective energy variation of the system is specified on a velocity basis rather than the absolute position of the acoustic source. Consequently, the designed system illustrates the localization output derived from the target displacement in order to conserve energy. According to the ISL, the node adjacent to the mobile sound source creates the PVVE output due to the exponential acoustic energy distribution. The PVVE algorithm only responds to the positive and negative transition created by significant exponential variation.

The CEB localization consolidates the individual acoustic energy from deployed nodes and estimates the target position by analyzing the contour distribution. The basic system architecture is simple and straightforward; however, the localization task requires a significant amount of energy without the algorithm extension for energy efficiency. Note that the persistent supervision of the entire nodes consumes considerable energy and time. An example of the algorithm is intelligent scheduling of the arranged nodes in order to improve the energy efficiency by predicting the forthcoming location of the target [14,16]. Most of the time, only the nodes around the target are awaiting the localization, and consequently the system presents certain energy efficiency by employing the complex algorithm and multi-level communications.

**Figure 1.**
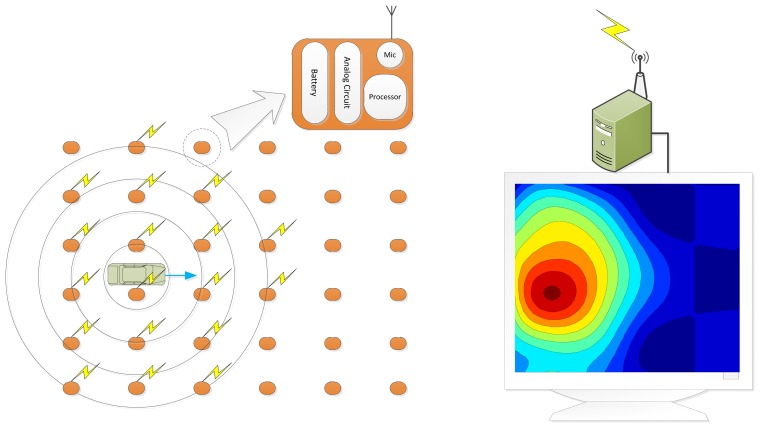
The functional diagram of the PVVE system.

The PVVE algorithm is simple and maneuverable for feasibility in terms of the computation and power consumption. Moreover, the complexity of the PVVE system is maintained as low for possible rapid deployment and integration with other systems in the field. The functional diagram of the PVVE system is as shown in Figure 1, which is identical to the CEB localization system; however, the energy distribution is related to the vehicle velocity. Note that the PVVE is situated in the individual node and the WSN acoustic localizer is extended to the PVVE system by simple modification at nodes and a collective analysis program.

For the solution of the given system, two consecutive filters in the PVVE such as the as analog envelope detector (ED) and digital exponential smoothing filter (ESF) present the velocity vector sensitive output. The cascade connection of the both filters operates mutually in a complementary sense in order to produce a zero output at a consistent signal level. However, the forward or backward exponential signal level from the moving target creates a transition output proportional to the speed. In addition to the low complexity of the filters, a prominent advantage of the system is its high feasibility for low energy configuration by initiating the communication based on the filter output. Only moving targets prompt further inter-node wireless talk besides multihop communications.

## 3. Methodology

The basis of PVVE is the specialized zero output filter for consistent level of input sound. The signal created by the stationary vehicle is relatively steady in magnitude; hence, the PVVE output rapidly converges to zero for a motionless source. The motion of the target represented by the velocity vector can be divided into the radial and azimuthal component in polar coordinates. The radial movement of a target around the PVVE node provides the natural variation of the sound level based on the Euclidean distance between the source and receiver. The significant level changes introduced by target motion near the node produce a transition PVVE output directly proportional to the radial velocity. The PVVE is designed to optimize the maximum transition response according to the corresponding intensity changes from the radial motions. In order to provide the operation method, this section presents the input signal model and overall PVVE filter architecture.

For the improved understanding of the system, three principal assumptions are employed regarding the acoustic signal and sound source. First, the signals arriving at the sensors are produced by the point source for isotropic propagation. Second, the sound level of the acoustic source is consistent in the temporal and spectral domains. Third, sources are located on a boundless place for the free field condition. With these assumptions, the sound energy propagates outwards spherically in an isotropic manner from a point source. The PVVE in the individual node assesses the energy distribution and variation in the time domain based on the measured signal. Note that the far field provision of the measurement requirement is released in this investigation; therefore, the wavefront of the propagation is allowed to be circular for near field sensing.

With designed preprocessing, the PVVE filter converts a measured sound level into the digital values for discrete computing. The value follows the natural distribution of sound level known as the ISL in a boundless and isotropic field. Due to the square relationship between surface area and radius of a sphere, the ISL justifies that the intensity of radiation passing through any unit area is inversely proportional to the square of the distance from the point source. The exponential relationship is illustrated in the following equation:

(1) $$L_{p}\left(r_{i}\right)=20\text{log}\left[\frac{a}{r_{i}-r_{0}}\right]\;\text{dB}$$

where *L_p_*(*r_i_*) is the sound pressure level in decibels at the distance *r_i_*. The terms *a* and *r*_0_ are model parameters for amplitude and distance, correspondingly.

**Figure 2.**
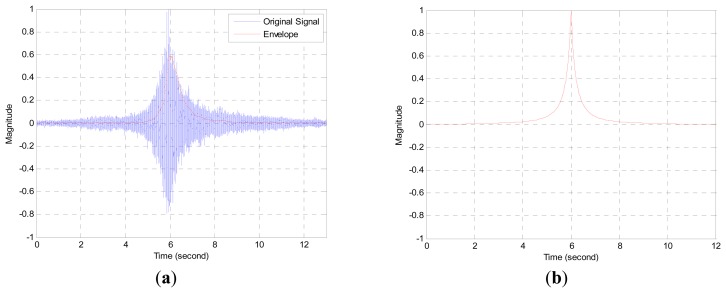
(**a**) Simulated field signal and corresponding envelope; (**b**) Simulated envelope from ISL (10 km/h and 100 Hz sampling frequency).

Along with the given equation, the magnitude demonstrates the significant variation around the proximity area in particular. Figure 2b is the derived and normalized plot from Equation (1) for a mobile target with 10 km/h speed and 100 Hz sampling frequency. At the time of six seconds, the target crosses the node and the received sound level increases and decreases exponentially in short time according to the ISL. With identical parameters, the simulated signal from the product between the measured and ISL of the mobile vehicle justifies the law in Figure 2a. Note that the envelope of simulated signal is obtained by the low pass filter from an infinite impulse response (IIR) system. The extreme level variation around the acoustic source is provided to the designated filter and the transition response of the filter is exploited extensively for the localization.

The velocity responsive filter is constructed from the configuration of the ESF which represents the IIR low pass filter. The ESF is the simple average filter between the current input *x*[*n*] and the previous output *y*[*n* − 1] with positive *β* parameter. The difference between the ESF output and input envelope is approximately proportional to the target velocity.

The high speed target illustrates the rapid exponential inputs to the ESF and the discrepancy between the rapid exponential input and smoothed filter output is expected to be significant. The relatively low speed target provides a gradual input to the ESF which delivers most of the smooth input to the output; therefore, the final output of the system is assumed to be trivial. The mathematical representation of the filter architecture is shown below:

(2) $$\begin{array}{c} y\left[n\right]=\left(1-\beta \right)x\left[n\right]+\beta y[n-1]\\ z\left[n\right]=x\left[n\right]-y[n] \end{array}$$

where *x*[*n*] is the input (sound envelope) to the system and *y*[*n*] is the output of the ESF. The *z*[*n*] is the system response which shows the difference between the ESP output and raw input *x*[*n*].

**Figure 3.**
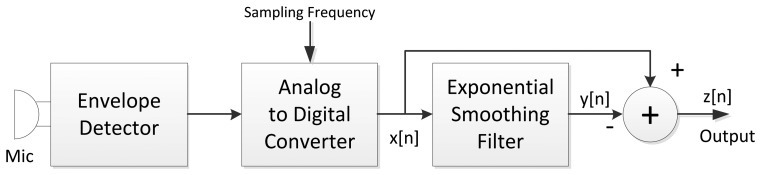
Individual node architecture.

The overall structure of the PVVE consists of three fundamental parts identified as ED, analog to digital converter (ADC), and ESF section. By using the low complex analog circuit, the ED efficiently extracts the average level of the sound in a continuous domain. Since the envelope of the sound dominantly provides the low frequency components, the ADC converts the signal to the discrete domain with low sampling frequency. The ESF section performs the operation given by Equation (2) with a simple microprocessor. The cascade connection between the explained parts is illustrated in Figure 3.

**Figure 4.**
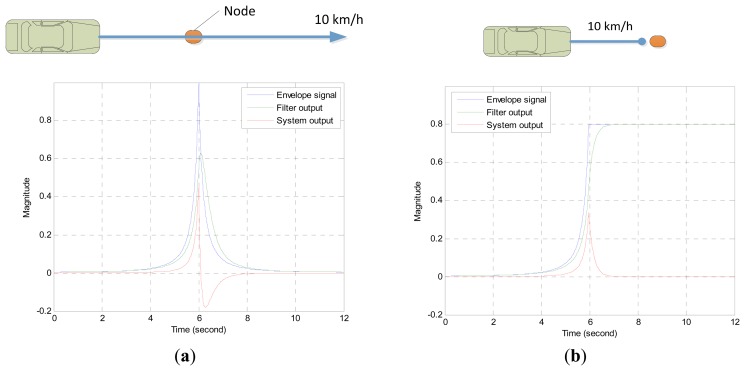
Example outputs for (**a**) a continuously moving vehicle and (**b**) a temporarily stopped vehicle.

The simulated outputs of the PVVE for two situations are demonstrated in Figure 4. The first situation shown in Figure 4a represents a constantly moving vehicle with 10 km/h velocity and a car directly crossing the PVVE node at the time of six seconds. The second scenario displayed in Figure 4b presents a roughly similar situation except for the stopping in front of the PVVE node. The constant moving case displays a very abrupt exponential distribution according to the ISL and the ESF produces the smoothed and delayed output plotted with a green line in Figure 4a. The PVVE output from the difference between the sound envelope and ESF output delivers the positive overshoot before the node and the negative undershoot afterwards due to the phased output of the ESF. Presumably, the height and depth of the shoot corresponds to the target velocity and the zero crossing indicates the vehicle passing over the node recently. The hypothesis will be verified in the next section with an analytical procedure.

The selective rejection feature of the PVVE is shown in Figure 4b for a temporarily stopped vehicle. The car stays in a stationary position in front of the node and continuously provides a constant sound level. From the given exponential step input, the ESF gradually follows a constant value with a certain phase shift. Therefore, the output of the PVVE exhibits a narrow peak and reverts to the null value subsequently as seen with the red line in Figure 4b. Compared with the CEB localization system, the PVVE yields a brief signal survival time for the halted situation; hence, the stationary target is discriminated from the further localization in order to conserve the deployed battery. Although not shown here, the sudden departure from the node proximity creates a negative valley in order to present the away vector of the vehicle. Basically, the negative shoot of the output is also derived from the phase difference between the direct input and ESF output as the other cases shown in Figure 4. As a result, the PVVE outputs exhibit a brief temporal length for all introduced situations:

(3) z[n]=β(x[n]−x[n−1]+z[n−1])

The discrete section of the PVVE is rearranged in Equation (3) denoting the first order IIR filter. The detailed derivation of the equation is given at Appendix A. The location of the pole and zero of the filter is *β* and one, respectively, and overall the filter shows a high pass filter. As the *β* approaches the position one, the filter demonstrates a sharper high pass filter. Note that the direct current gain is constantly zero because of the zero location of the filter unless the value of *β* is one. The complete PVVE consists of the ED (low pass filter) and the discrete section (high pass filter) in cascade connection and performs a zero output filter due to the inverse relationship between filters. Consequently, the output of the PVVE is transitional and most likely transient.

## 4. Analysis

**Figure 5.**
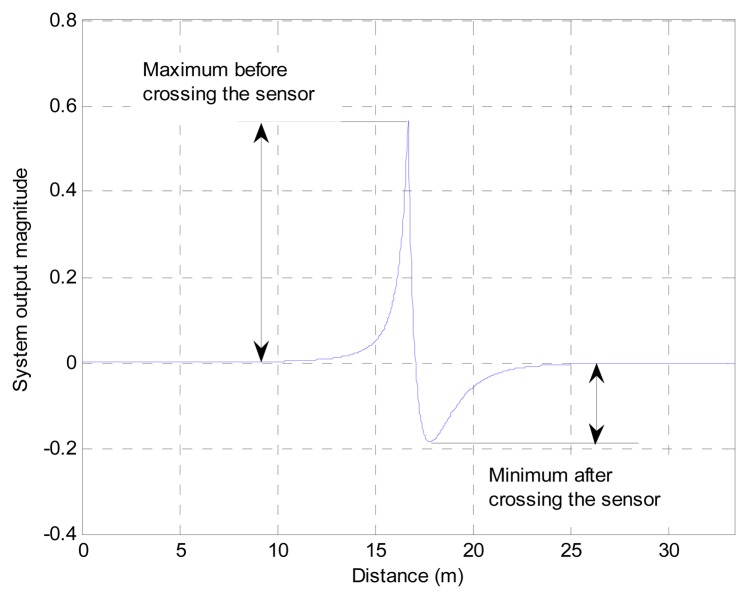
Performance criterion (maximum and minimum) of the PVVE output.

This section presents the analytical procedure in order to decide the optimal *β* value in the PVVE. The maximum and minimum values across the zero crossing are employed and shown in Figure 5. The further outstanding peak and valley are expected to provide an improved localization due to the high sensitivity of the output against the target velocity. The range of the *β* value is from zero up to one and the analytical procedure determines the coefficient which offers the prominent output. We observe that the time span of the PVVE is proportional to the height and depth; therefore, the evaluation considers magnitude only.

Instead of using the sampling frequency and vehicle velocity individually, the combined variable called sampling distance (SD) is adopted for assessment. The mathematical representation is given by Equation (4):

(4) $$s_{d}=\frac{1}{f_{s}}\left(v_{t}\frac{1000}{3600}\right)$$

where *f_s_* is the sampling frequency in Hz and *v_t_* is the vehicle velocity in km/h. The units of SD are meters. The high velocity shows the coarse SD and increased sampling frequency presents the dense SD according to the equation. The ratio between the velocity and sampling frequency as *v_t_*/*f_s_* covers from the 0.001 up to 1 in this analysis and the corresponding SD is from 2.778 × 10^−4^ up to 0.2778.

**Figure 6.**
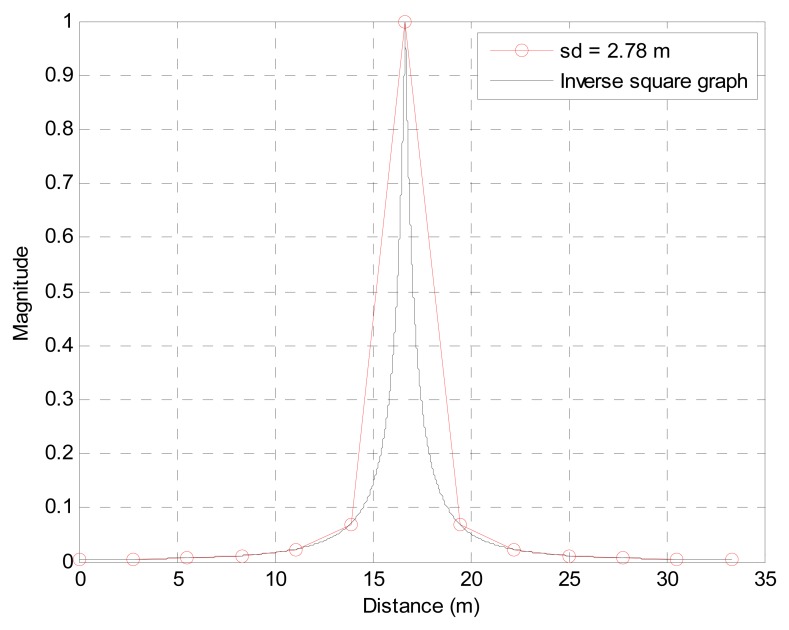
Example of sampling distance (target velocity = 10 km/h and sampling frequency = 1 Hz).

The SD example is illustrated in Figure 6 for 10 km/h velocity and 1 Hz sampling. The black solid line in the plot describes the ISL in a continuous domain and the red circles depict the sampled location from the PVVE node based on the given parameters. Due to the high SD, discrete sampled data after ADC demonstrate a rapid increase and decreasing data for the filter. On the other case, the low SD densely obtains the data which presents a relatively gradual variation. In the next subsections, mathematical and analytical procedures deliver the optimal *β* value and relationship between the PVVE output and velocity of the target over the given SD range.

### 4.1. Case for an Approaching Target

**Figure 7.**
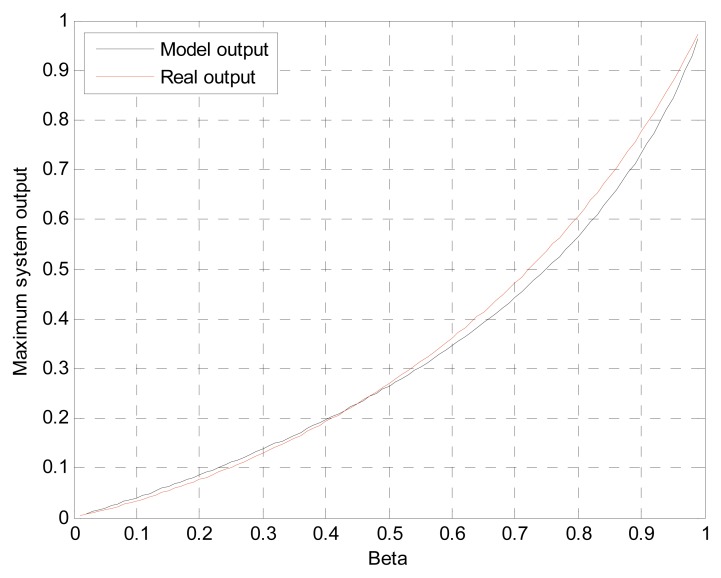
Comparison graph of PVVE output for the mathematical model and simulated real output.

An approaching target provides the situation to create the exponentially increasing input to the PVVE node. Before the target intersects the node, the filter acquires the rapid growing input and produces the consequent output based on the target velocity, sampling frequency and *β* value. The mathematical modeling of the circumstances is derived at Appendices B and C and the outcome is shown below:

(5) $$\begin{array}{l} z\left[N\right]=\beta -\left(1-\beta \right)\frac{f_{s}^{2}}{v_{t}^{2}}C\beta^{-1}\left(Li_{2}\left(\beta \right)-\beta \right)\\ \;=\beta -\left(1-\beta \right)\frac{f_{s}^{2}}{v_{t}^{2}}C\beta^{-1}\left(-\int_{0}^{1}\frac{\ln(1-\beta t)}{t}\text{d}t-\beta \right) \end{array}$$

where the *v_t_* is target velocity and *f_s_* is sampling frequency. Also, *z*[*N*] is the PVVE output (or maximum system output in the approaching situation) with arbitrary constant *C* at the present time *N* and *Li*_2_(*β*) is the dilogarithm. Notice that the equation is developed from the normalized magnitude and infinite length of the excitation signal. Since no simple closed-form equation exists for the dilogarithm, the alternative numerical approach with integral [31] is employed for the last part of Equation (5). Figure 7 illustrates the two graphs from the mathematical model and simulation outcome for evaluation purposes. According to the plot, the mathematical model from Equation (5) properly represents the system output with the marginal difference caused by the temporal scope discrepancy.

The improved localization originated from the prominent output of the PVVE and higher response of the filter provides a better performance for the approaching target case. From the equation and plot, the filter output is direct proportional to the *β* value; hence, near one *β* is expected to show the outstanding output from the filter.

**Figure 8.**
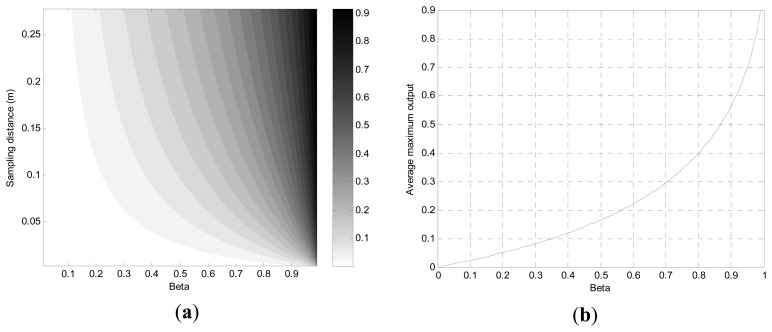
(**a**) Maximum system output distribution in terms of *β* and SD; (**b**) Averaged maximum system output over SD in terms of *β*.

Figure 8a demonstrates the maximum system output in terms of SD and *β* simultaneously by using the Equation (5). The figure shows that the upper right corner represents the elevated output and the opposite direction (lower left corner) exposes the diminished response from the PVVE. With fixed sampling frequency, the upper right area can be reached by the increased velocity with near one *β* value; therefore, the proportional relationship between the PVVE output and the target velocity is justified by the analytical figure. Figure 8b organizes the discovered correlation by representing the average plot of the Figure 8a over the vertical axis as well. Note that the Equation (5) may cause a misleading view of the relationship due to the denominator location of the velocity. In the equation, the second term which includes the *v_t_* is always negative; therefore, the higher velocity subtracts the lesser value from the *β* and shows the increased PVVE output as a result.

In the approaching target scenario, the graph and equation display the proportional relationship between the *β* values and filter output without any global and local maxima. The absence of the maxima provides the optimal solution near the boundary region of the *β* value; hence, the higher *β* is preferred for localization. The specific *β* value is determined by the further analysis from the next subsection for the withdrawing target situation in which a negative valley is produced after target passes by. The deeper valley with higher peak based on the optimal *β* value is determined and provided for improved localization.

### 4.2. Case of a Withdrawing Target

The withdrawing vehicle presents the case to generate the exponentially decreasing input to the PVVE node. After the target crosses the node, the filter obtains the quickly decreasing input according to the ISL and provides the corresponding response based on the given parameters. The mathematical modeling of the situation is derived in Appendix B and C and the result is shown below:

(6) $$z\left[N\right]=-\left(1-\beta \right)\frac{f_{s}^{2}}{v_{t}^{2}}C\underset{N\to \infty }{\text{lim}}\beta^{N}\left(\sum_{q=1}^{N-1}\frac{{\left(\beta^{-1}\right)}^{q}}{q^{2}}-\beta^{-1}\right)$$

where the *v_t_* is target velocity and *f_s_* is sampling frequency. Also, *z*[*N*] is the PVVE output (or minimum system output in the withdrawing situation) with arbitrary constant *C* at the present time *N*. By using the dilogarithm, further simplification of the equation cannot be performed in Equation (6) due to the range of the *β* values which yields a dilogarithm diverging to infinity. The direct analysis of the sensitivity over the *β* value is barely possible in the given equation because of the complex high order series combination.

**Figure 9.**
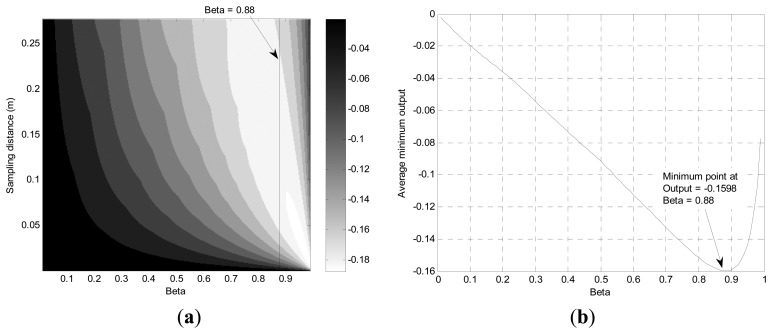
(**a**) Minimum system output distribution in terms of *β* and SD; (**b**) Averaged minimum system output over SD in terms of *β*.

The simulation based on Equation (3) and ISL input signal is performed and arranged for minimum system output under various conditions in Figure 9. For outstanding output of the PVVE, the lower minimum output of the filter gives the enhanced operation for the withdrawing vehicle situation. From Figure 9a, the distribution of the minimum output shows a parabolic profile with a downward concave curve in horizontal perspective for most of the SD range.

With the given gray map in Figure 9a, the whiter hue presents the lower output from the PVVE for the withdrawing situation. Compared with approaching target case, the optimal values (white area) are located at the off boundary region because the distribution shows no global proportionality between the minimum output and *β* values. Over the given range of the SD, the averaged minimum output is depicted in Figure 9b. The minimum output is diminished linearly up to near 0.9 in *β* and increased rapidly thereafter in the figure. The discovered optimal output values from the simulation are 0.88 in *β* and −0.1598. The value of 0.88 in *β* complies favorably with the request generated by the previous situation as the higher *β* is preferable. Consequently, the selected *β* value is adopted for the PVVE for the subsequent investigations.

## 5. Results

The previous sections present the computer experiments and mathematical study with a single node in order to derive the optimal filter parameter based on the ISL input. This section provides collective simulations over virtually deployed nodes for the specific situations along with the field ISL parameters. However, the overall quantitative analysis in terms of energy usage cannot be achieved in this paper due to the extensive spectrum of the vehicle movement scenario. Instead, a qualitative investigation is performed for two moving situations by employing time-lapse figures; therefore, the general benefit of the PVVE system is described by the energy distribution compared with the CEB localization system. Also, the computational requirement for the PVVE is developed to illustrate the low complexity of the algorithm.

**Figure 10.**
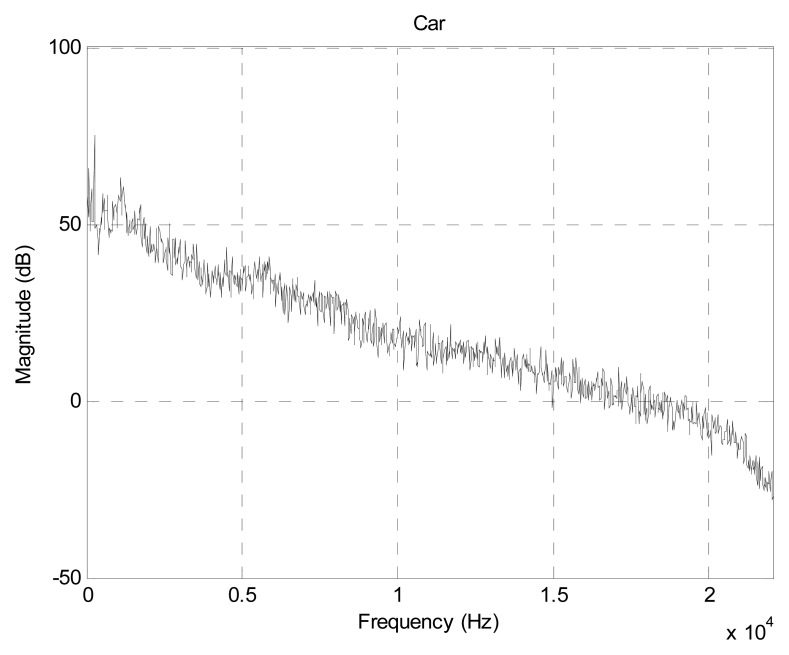
Spectrum of an automobile.

To evaluate the PVVE system, the realistic acoustic data representing the conventional situation is required for further analysis. The general sound from an outdoor field is most likely to change in magnitude and spectrum over time; therefore, the stationary spectrum is devised to execute the simulations in order to derive a consistent outcome. The selected sound source is recorded at the fixed location over the moving object. The range of time data that corresponds to the clear representation of acoustic characteristic is used as the FIR filter coefficient for generating the stationary sound signal based on the white input signal. By utilizing the given procedure, the produced sound can be manipulated for any data set length of in order to provide sufficient simulation time. Figure 10 displays the spectrum plot characterized by extensive distribution of the power over the wide frequency range.

**Figure 11.**
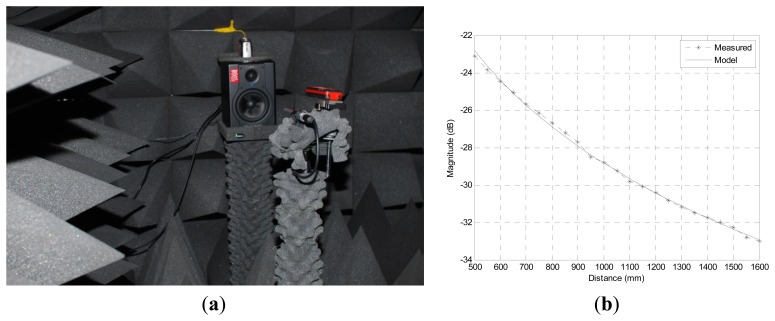
(**a**) Experiment and (**b**) distribution of sound pressure level in the anechoic chamber.

The volume of acoustic source is normalized at the time of wave creation and the received magnitude at individual nodes is determined by the ISL and Euclidian distance between the source and receiver. For precise simulation, the ISL parameters from Equation (1) are estimated by experimental practice in a proper environment. The spatial exploration is executed and analyzed in the anechoic chamber shown in Figure 11a which has been verified to exhibit partial conformity with ISO 3745 for free field and hemi-free field conditions [32]. The devised sound generated from one end of the free field chamber and microphone moves along the paths for each test signal starting 50 cm away from the speaker and ending at the chamber wall with 5 cm apart spacing. With the recorded sound magnitude, the least square methods over the linearized Equation (1) computes the optimal parameters *a* = 36.1438 and *r*_0_ = 0.0043. The measured and modeled data are illustrated in Figure 11b with maximum deviation of 0.292 dB.

**Figure 12.**
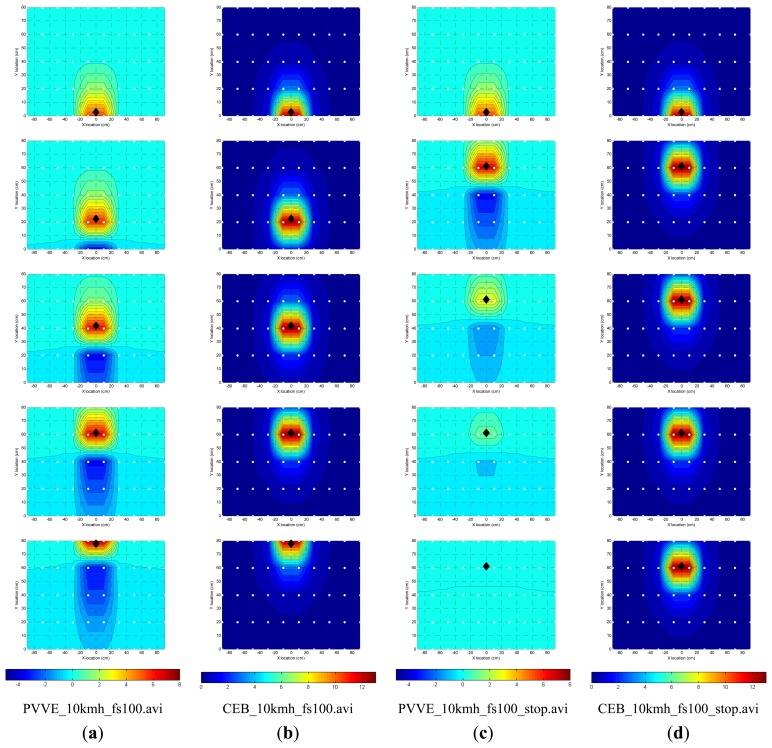
Snapshots of energy distribution for a moving target (black diamonds) at 10 km/h with 100 Hz sampling frequency over 50 nodes (white dots). Constantly moving for PVVE (column **a**) and CEB (column **b**) localization system. Temporarily stopped for PVVE (column **c**) and CEB (column **d**) localization system. The last row represents the video file names attached to this paper for each system and situation.

For the simulation, a total of 50 nodes are deployed over the reconnaissance field every 20 cm apart in the vertical and horizontal direction and the acoustic target is assumed to be a point source under free field conditions. Based on the estimated parameters, the magnitude information for each node is calculated and employed over the normalized acoustic sound source. The processed sound is measured information for the individual node and the receiver executes the PVVE operation according to the system configuration given by Figure 3. In general, the ED in the system is implemented by the simple analog circuit but is realized by the discrete IIR filter for the computer simulation purposes. The CEB localization system transmits the direct output of the ED to the front-end station in order to find the target coordinates via the spatial energy distribution. The PVVE node sends the upshot of the additional process identified as zero output filter for the localization procedure. Figure 12 shows the time-lapse images of energy distribution for the CEB and the PVVE localizer in two situations: constantly moving and temporarily stopped. Figure 12a (first column) and Figure 12b (second column) denote the instance of constant moving for the PVVE and CEB localization system respectively. Also, Figure 12c (third column) and Figure 12d (last column) stand for the temporarily stopped circumstance for the PVVE and CEB localization system, correspondingly. The consistently moving situation in Figure 12a and Figure 12b is illustrated by five images in rows and the temporal direction elapsed in the downward direction from the top row. The difference between successive images is seven samples in time and 19.4 cm in distance for 100 Hz sampling frequency and a 10 km/h moving target.

The CEB localization system exhibits a positive hill along with target location and the PVVE system presents a positive hill as well as the negative valley right after target trail. Note that the color maps of the PVVE and CEB localization system are not identical and illustrated in the second to last row. Both systems can discover the target coordinates by estimation of the positive center from the contour distribution. The shape of the hill in the PVVE system displays the asymmetric property in the movement direction and zero crossing indicates that the vehicle starts to withdraw from the node. Besides the negative valley in the PVVE system, no significant difference between systems can be observed in the constant movement situation.

The last two columns in Figure 12 specify the energy distributions for the temporarily stopped case. In each system, the difference between consecutive pictures is 21 samples in time and 58.2 cm in distance with unchanged configuration from the previous circumstance. Up to the second row, the target moves to the upper direction consistently and then stays in the last position steadily. Therefore, from the third to last row of the two rightmost columns, the figures indicate the motionless target over the deployed field. The CEB localization system illustrates identical energy distributions for the stationary target and the system requires continuous communications between nodes and the front-end station for localization and decision. The PVVE system provides gradually decreasing distributions in a timely manner and the PVVE node autonomously avoids further communications for a static target. In the given situation, the stationary vehicle completely disappears from the distribution after 63 samples or 0.63 s in the time window. The last row of Figure 12 shows the file names for the video which consolidate the continuous pictures of energy distribution without time adjustment. Notice that the files can be found with this *Sensors* paper on the MDPI website and it is recommended they be played with the VLC media player.

The principal advantage of the PVVE system is that the individual node determines the target status with simple computation and prevents unnecessary communications with the stationary target. The front-end station requires some additional workload in order to maintain the disappeared targets in the surveillance field. In general, the station contains an extra capacity energy source with high performance computing resources; therefore, the lock-on algorithm on the station can be performed fluently without reducing the system operability. In a WSN system, one of the major factors for sustainability is the energy consumption from the deployed nodes which have limited power resources for rapid and versatile operations. Excluding the sensing hardware, the significant energy consumers in the node are the computing processor and communication devices. The relationship in energy consumption between the communication and computation is heavily dependent on the particular hardware in use; however, the energy requirement ratio of one bit of communication to one computation instruction typically ranges from several hundreds to thousands [33,34]. Due to the excessive energy requirements for communication, the WSN system is advanced in one of the ways to avoid redundant transmission of the information. Nevertheless, the progression is limited by the low computing power of the node which hardly provides enough capability to perform the preprocessing which is censoring the communication.

The advantage of the PVVE algorithm denotes the low complexity in computation which can be performed by a conventional low power microprocessor. Table 1 organizes the pseudo-code and the computational requirements of PVVE digital section. The abstract level code shown above is written in reduced instruction set computing (RISC) instructions which are compatible with general microprocessors. In general, the RISC processor classifies the instructions into data transfer operations, arithmetic & logical operations, and branching operations. The devised code considers the data transfer as *M*, simple arithmetic as 1, and complex arithmetic as *N* cycles of processor clock time. Total execution time is 4*M* + 2*N* + 3 cycles except for the branching operation which is highly dependent on the processor architecture and implementation. One example of a numeric execution time for a loop body is a cycle time of 15 (*M* = 2 and *N* = 2) for the Atmel AVR ATmega16(L) microprocessor. Most conventional microprocessors shows less than several dozens of cycles for PVVE algorithm execution with a branching operation. According to the communication and computation ratio for energy, the PVVE system reduces the energy consumption from a tenth up to a hundredth compared to the CEB localization which uses frequent communication. Hence, the stationary target situation illustrates the significant energy improvement possible with the PVVE system.

## 6. Conclusions

This paper presents a novel lightweight filter architecture for mobile vehicle localization over WSNs. The node filter consists of an ED and high pass filter connected in cascade for a zero output filter named PVVE which responds to the velocity vector to the node. For a mobile vehicle, the significant level change introduced by the ISL produces the PVVE transition output and the collective energy distribution from the front-end station localizes the target by analyzing the contour plot. However, the stationary target yields zero converging output from the PVVE and the node autonomously prevents the further communications which is an expensive operation in terms of energy usage. The front-end station requires the additional workload to maintain a motionless target in the monitoring field; on the other hand, the deployed node conserves considerable energy by avoiding unnecessary communications which deliver the energy from a fixed position target. For the improved understanding of the system, the principal assumptions on the acoustic signal and sound source as point source, isotropic propagation, consistent source in time and frequency, and free field are employed. Based on the assumptions, our mathematical investigation provides the optimal PVVE parameter that produces the prominent transition output. The collective simulation illustrates significant energy savings for the stationary target situation compared with the CEB localization system. The PVVE system reduces the significant amount of energy by performing simple computations rather than expensive communication. The complexity of the algorithm is verified by the RISC instructions for the PVVE algorithm in order to derive the approximate execution cycles.

The PVVE system proposed in this paper presents a novel distributed decision system for mobile target localization based on WSNs. The conventional system generally requires information collection which consumes considerable energy and time. The PVVE system hands the partial decision process to the field node and reduces the energy usage by eliminating further data transfer caused by stationary targets. The low complexity of the PVVE algorithm is beneficial for improving the feasibility of the system with a low power microprocessor. Future work will involve research on the quantitative analysis of the PVVE system for various target movement situations in terms of energy usage. Moreover, a realistic data set from loose assumptions can be applied to the PVVE system for performance analysis over a real field. The final objective of our PVVE system studies in the near future is field deployed experiments with optimal configuration and various vehicles.

## Figures and Tables

**Table 1.** Pseudo-code of PVVE algorithm (digital section only).

**Pseudo-code**	**# of clock cycles**
*Loop starts*	
*Load x*[*n*] *to register*	*M*
*Load y*[*n* − 1] *to register*	*M*
*Register multiplication to perform* (1 − *β*)*x*[*n*]	*N*
*Register multiplication to perform βy*[*n* − 1]	*N*
*Register addition to perform y*[*n*] *=* (1 − *β*)*x*[*n*] *+ βy*[*n* − 1]	1
*Store y*[*n*] *to memory*	*M*
*Register subtraction to perform z*[*n*] *= x*[*n*] − *y*[*n*]	1
*Store z*[*n*] *to memory*	*M*
*Change n value*	1
*Loop ends*	

**Figure A1.**
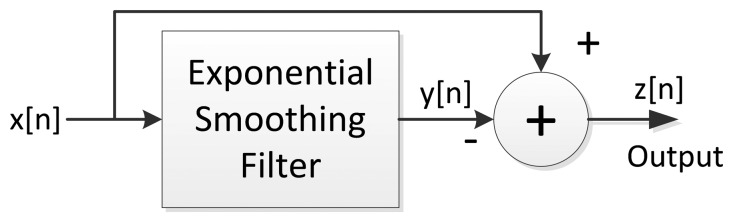
Filter configuration.

**Figure C1.**
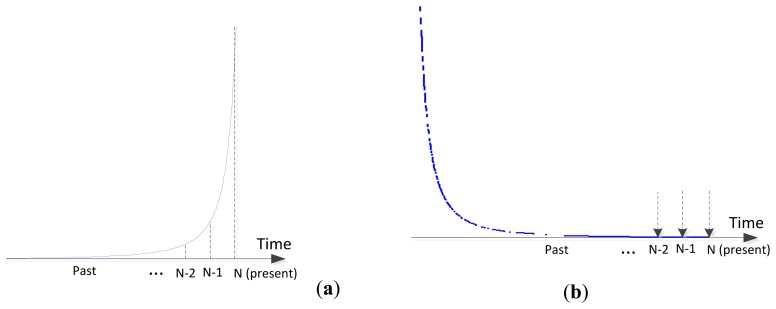
Input signal according to ISL for target (**a**) approaching and (**b**) withdrawing case.

## Appendix

### A. Equation Derivation for the Discrete Section of PVVE

Note that 0 < *β* < 1, and ESF is given as below

y[n]=(1−β)x[n]+βy[n−1]

The system output is computed based on the subtraction between the input and output of the filter.

z[n]=x[n]−y[n]=x[n]−(1−β)x[n]−βy[n−1]=βx[n]−βy[n−1]

Since *z*[*n*]=*x*[*n*] − *y*[*n*], therefore *y*[*n* − *1*] = *x*[*n* − 1] − *z*[*n* − 1]

z[n]=βx[n]−βy[n−1]=βx[n]−β(x[n−1]−βz[n−1])=β(x[n]−x[n−1]+z[n−1])

Therefore,

z[n]=β(x[n]−x[n−1]+z[n−1])

## B. Closed-Form Equation for the Discrete Section of the PVVE

The ESF is given as below

y[n]=(1−β)x[n]+βy[n−1]

$$\begin{array}{l} y[n]=\left(1-\beta \right)x\left[n\right]+\beta \left\{\left(1-\beta \right)x\left[n-1\right]+\beta y\left[n-2\right]\right\}\\ \;=\left(1-\beta \right)x\left[n\right]+\beta \left(1-\beta \right)x\left[n-1\right]+\beta^{2}y\left[n-2\right]\\ \;=\left(1-\beta \right)x\left[n\right]+\beta \left(1-\beta \right)x\left[n-1\right]+\beta^{2}\left\{\left(1-\beta \right)x\left[n-2\right]+\beta y\left[n-3\right]\right\}\\ \;=\left(1-\beta \right)x\left[n\right]+\beta \left(1-\beta \right)x\left[n-1\right]+\beta^{2}\left(1-\beta \right)x\left[n-2\right]+\beta^{3}y\left[n-3\right]\\ \;=\left(1-\beta \right)x\left[n\right]+\beta \left(1-\beta \right)x\left[n-1\right]+\beta^{2}\left(1-\beta \right)x\left[n-2\right]+\beta^{3}\left(1-\beta \right)x\left[n-3\right]+\beta^{4}y\left[n-4\right] \end{array}$$

Generalize the *y*[*n*]

$$y[n]=\left(1-\beta \right)x\left[n\right]+\left(1-\beta \right)\sum_{k=1}^{n-2}\beta^{k}x\left[n-k\right]+\beta^{n-1}y\left[1\right]$$

Therefore, the system output *z*[*n*] is given as below equation.

$$z\left[n\right]=x\left[n\right]-y\left[n\right]=\beta x\left[n\right]-\left(1-\beta \right)\sum_{k=1}^{n-2}\beta^{k}x\left[n-k\right]-\beta^{n-1}y\left[1\right]$$

## C. Equation Derivation for the Approaching and Withdrawing Situation

### Target Approaching Case

The distance between target and node at the discrete time of *n* is

$$r\left[n\right]=\frac{v_{t}}{f_{s}}(N-n+1)$$

where the *v_t_* is target velocity and *f_s_* is sampling frequency. The range of *n* is from one up to *N* (present) and the input magnitude *x*[*n*] is corresponding to the ISL over the distance *r*[*n*]

$$x\left[n\right]=\frac{C}{r^{2}[n]}=C{\left[\frac{f_{s}}{v_{t}(N-n+1)}\right]}^{2}$$

*C* is an arbitrary constant. The system output at present *N* from the Appendix B is

$$\begin{array}{l} z\left[N\right]=\beta x\left[N\right]-\left(1-\beta \right)\sum_{k=1}^{N-2}\beta^{k}x\left[N-k\right]-\beta^{N-1}y\left[1\right]\\ \;=\beta x\left[N\right]-\left(1-\beta \right)\sum_{k=1}^{N-2}\beta^{k}C{\left[\frac{f_{s}}{v_{t}\left(k+1\right)}\right]}^{2}-\beta^{N-1}y\left[1\right]\\ \;=\beta x\left[N\right]-\left(1-\beta \right)\frac{f_{s}^{2}}{v_{t}^{2}}C\sum_{k=1}^{N-2}\frac{\beta^{k}}{{\left(k+1\right)}^{2}}-\beta^{N-1}y\left[1\right] \end{array}$$

Substitute *q* = *k* + 1,

$$\sum_{k=1}^{N-2}\frac{\beta^{k}}{{\left(k+1\right)}^{2}}=\sum_{q-1=1}^{N-2}\frac{\beta^{q-1}}{q^{2}}=\beta^{-1}\sum_{q=2}^{N-1}\frac{\beta^{q}}{q^{2}}=\beta^{-1}\left(\sum_{q=1}^{N-1}\frac{\beta^{q}}{q^{2}}-\beta \right)$$

Let's assume that the maximum input value *x*[*N*] is one and the initial ESF output value *y*[1] is zero

$$z\left[N\right]=\beta x\left[N\right]-\left(1-\beta \right)\frac{f_{s}^{2}}{v_{t}^{2}}C\beta^{-1}\left(\sum_{q=1}^{N-1}\frac{\beta^{q}}{q^{2}}-\beta \right)-\beta^{N-1}y\left[1\right]=\beta -\left(1-\beta \right)\frac{f_{s}^{2}}{v_{t}^{2}}C\beta^{-1}\left(\sum_{q=1}^{N-1}\frac{\beta^{q}}{q^{2}}-\beta \right)$$

Considering the infinite long previous values to compute the present system output *z*[*N*]

$$\underset{N\to \infty }{\text{lim}}z\left[N\right]=\beta -\left(1-\beta \right)\frac{f_{s}^{2}}{v_{t}^{2}}C\beta^{-1}\underset{N\to \infty }{\text{lim}}\left(\sum_{q=1}^{N-1}\frac{\beta^{q}}{q^{2}}-\beta \right)=\beta -\left(1-\beta \right)\frac{f_{s}^{2}}{v_{t}^{2}}C\beta^{-1}\left(Li_{2}\left(\beta \right)-\beta \right)$$

*Li*_2_(*β*) is dilogarithm (also known as Spence's function) as below

$$Li_{2}\left(\beta \right)=\sum_{k=1}^{\infty }\frac{\beta^{k}}{k^{2}}$$

There is no closed-form solution for the dilogarithm but the numerical solution is given as below [31]

$$Li_{2}\left(\beta \right)=-\int_{0}^{\beta }\frac{\ln(1-t)}{t}dt=-\int_{0}^{1}\frac{\ln(1-\beta t)}{t}\text{d}t$$

Finally,

$$z\left[N\right]=\beta -\left(1-\beta \right)\frac{f_{s}^{2}}{v_{t}^{2}}C\beta^{-1}\left(-\int_{0}^{1}\frac{\ln(1-\beta t)}{t}dt-\beta \right)$$

#### Target Withdrawing Case

The distance between target and node at the discrete time of *n* is

$$r\left[n\right]=\frac{v_{t}}{f_{s}}n$$

$$x\left[n\right]=\frac{C}{r^{2}[n]}=C{\left[\frac{f_{s}}{v_{t}n}\right]}^{2}$$

The current system output is

$$\begin{array}{l} z\left[N\right]=\beta x\left[N\right]-\left(1-\beta \right)\sum_{k=1}^{N-2}\beta^{k}x\left[N-k\right]-\beta^{N-1}y\left[1\right]\\ =\beta x\left[N\right]-\left(1-\beta \right)\sum_{k=1}^{N-2}\beta^{k}C{\left[\frac{f_{s}}{v_{t}\left(N-k\right)}\right]}^{2}-\beta^{N-1}y\left[1\right]\\ \;=\beta x\left[N\right]-\left(1-\beta \right)\frac{f_{s}^{2}}{v_{t}^{2}}C\sum_{k=1}^{N-2}\frac{\beta^{k}}{{\left(N-k\right)}^{2}}-\beta^{N-1}y\left[1\right] \end{array}$$

Substitute *q* = *N* − *k*

$$\sum_{k=1}^{N-2}\frac{\beta^{k}}{{\left(N-k\right)}^{2}}=\sum_{N-q=1}^{N-2}\frac{\beta^{N-q}}{q^{2}}=\beta^{N}\sum_{-q=1-N}^{N-2-N}\frac{\beta^{-q}}{q^{2}}=\beta^{N}\sum_{q=2}^{N-1}\frac{\beta^{-q}}{q^{2}}=\beta^{N}\sum_{q=2}^{N-1}\frac{{\left(\beta^{-1}\right)}^{q}}{q^{2}}=\beta^{N}\left(\sum_{q=1}^{N-1}\frac{{\left(\beta^{-1}\right)}^{q}}{q^{2}}-\beta^{-1}\right)$$

Let's assume that the current input value *x*[*N*] is zero and the initial ESF output value *y*[1] is zero

$$\begin{array}{c} z\left[N\right]=\beta x\left[N\right]-\left(1-\beta \right)\frac{f_{s}^{2}}{v_{t}^{2}}C\beta^{N}\left(\sum_{q=1}^{N-1}\frac{{\left(\beta^{-1}\right)}^{q}}{q^{2}}-\beta^{-1}\right)-\beta^{N-1}y\left[1\right]\\ \;=-\left(1-\beta \right)\frac{f_{s}^{2}}{v_{t}^{2}}C\beta^{N}\left(\sum_{q=1}^{N-1}\frac{{\left(\beta^{-1}\right)}^{q}}{q^{2}}-\beta^{-1}\right) \end{array}$$

Considering the infinite long previous values to compute the present system output *z*[*N*]

$$\underset{N\to \infty }{\text{lim}}z\left[N\right]=-\left(1-\beta \right)\frac{f_{s}^{2}}{v_{t}^{2}}C\underset{N\to \infty }{\text{lim}}\beta^{N}\left(\sum_{q=1}^{N-1}\frac{{\left(\beta^{-1}\right)}^{q}}{q^{2}}-\beta^{-1}\right)$$

Finally,

$$z\left[N\right]=-\left(1-\beta \right)\frac{f_{s}^{2}}{v_{t}^{2}}C\underset{N\to \infty }{\text{lim}}\beta^{N}\left(\sum_{q=1}^{N-1}\frac{{\left(\beta^{-1}\right)}^{q}}{q^{2}}-\beta^{-1}\right)$$
